# Supplementary material for: Patterns of Intron Gain and Loss in Fungi
Source: PLoS Biol. 2004 Nov 30;2(12):e422. doi: 10.1371/journal.pbio.0020422 (PMC532390; doi:10.1371/journal.pbio.0020422)
Supplement: Table S1 — Also available at http://genes.mit.edu/NielsenEtAl/. (4.3 MB ZIP). [file pbio.0020422.st001.zip › NielsenEtAl/html/1247.html]

AN3421.1.NCU04933.1.MG09333.1.FG08465.1


```
 CLUSTAL W (1.82) Multiple Sequence Alignments - Introns Inserted


Sequence 1: NCU04933.1	277 aa
Sequence 2: FG08465.1	249 aa
Sequence 3: MG09333.1	273 aa
Sequence 4: AN3421.1	249 aa
Alignment Length: 301 aa
Number Identitical Residues: 66 aa
Alignment Score (without introns) 3777


MG09333.1 	MPKSAALSIGRCVLLLR2LVTC1ATGLVGSAVLDAMLSNPS--ITKITILSRRPVAQATA
NCU04933.1	--------------MHL~ILTG~ATGLIGSAVLDAMIKTKD--ITKISIISRRPVKMAED
FG08465.1 	--------------MHL~ILTG~ATGLVGSSVLDAMLKSKE--ITKISILSRRPVPLAE-
AN3421.1  	--------------MRL~ILTG~ATGVVGSAALNHILSLPAGEISALYIISRSPVPAAE-
          	              :   ::*  ***::**:.*: ::.   ..*: : *:** **  *  

MG09333.1 	RADPRVSVVLHDDFTSYDDAALRNRLQDARGCVWALGISQTQVGSRD~EYERITKTFALA
NCU04933.1	AKDPRINVILHKDFEKYDSELL-NQLQGATGCVWALGISQNAVG-KE2EYVKITKDFPLA
FG08465.1 	-KDSRVNTIIHKDFTKYEPKVL-EQLQGANGVVWALGISQLKVT-KE2EYVTITRDFPVA
AN3421.1  	-NKPNVTVIQHTNYNEYPQELL-DKLKGADGCIWAQGISQTLVT-KE2EYIKITLDYPLA
          	  ...:..: * :: .*    * ::*:.* * :** ****  *  :: **  **  :.:*

MG09333.1 	AARSFGDVARDNG---------RPFDFVYVSGLG~ATTKPGR--------FAQLFARVKG
NCU04933.1	AAAAFQTLPPSSSSSPSPDQDKEPFRFIYVSGEG~TTTTPPSSQSPLSRLTTPFFAIIKG
FG08465.1 	AAEALSTLPPAN----------EPFRFIYVSAYG~ATTTPGT--------FTSLFGRVKG
AN3421.1  	AAKAFSTLS-------------DSFNFVYVSGEG0ATQTPGM--------FTPLFGRVKG
          	** ::  :.              .* *:***. * :* .*           : :*. :**

MG09333.1 	EAELGLAALQDENPLLRASTVRAGFIDTAAH-EAVKEYIPAK---PILTNLTEMVLAPVV
NCU04933.1	QAELALSQLS--TSTFLPFSMRPAFIDAAAH-PAIHPYIPKV---SGLYTLSNCVLGPPI
FG08465.1 	EAERKLSELR--TSKFHVETVRPCHVDAANH-DAIKPHIPSP---GIVYNALDLVFGPIT
AN3421.1  	QAETALLNLSTKYPSLKPFCVRPAYVDTANDPETLKARLRRPDQQSFKAKSIHAIFGPLM
          	:**  *  *  . . :    :*. .:*:* .. :::  :   ...    .  . ::.*  

MG09333.1 	RGFWKSAWSPTEHLGKFLTDMAVGRWSEKEMVA---AGAEKIG--KGGFLVVENTVFRKL
NCU04933.1	RAGYKKMWSPTEHLGKFLVEMAMGKWDETIRKEAEVSNGKGFEKLEGGSVVVGNEGWRRI
FG08465.1 	RTLLHSMHSPTERLGPFLTEMAMGKYDKQLD-----AGGNGISNLNG-SRILENVAFQRL
AN3421.1  	RNVLTKSDSPTEHLGKFLTDLAKGDGK--------PLSGEGVA---GGGRIIPNVVFRRV
          	*    .  ****:** **.::* *  .          ..: .    *.  :: *  ::::

MG09333.1 	QGLNA
NCU04933.1	ASLEG
FG08465.1 	YRP--
AN3421.1  	AGI--
          	
```
